# Contribution of diversity of social participation on the mental health of humanitarian migrants during resettlement

**DOI:** 10.1017/S2045796024000313

**Published:** 2024-05-23

**Authors:** Weiqing Jiang, Yuwei Yang, Yitong He, Qianyu Liu, Xueqing Deng, Yilin Hua, Alimila Hayixibayi, Yanyan Ni, Lan Guo

**Affiliations:** 1 Department of Medical Statistics and Epidemiology, School of Public Health, Sun Yat-sen University, Guangzhou, People’s Republic of China; 2 Institute of Infectious Disease Control and Prevention, Guangdong Provincial Center for Disease Control and Prevention, Guangzhou, People’s Republic of China; 3 School of Psychology and Counselling, Queensland University of Technology, Brisbane, QLD, Australia; 4 LKS Faculty of Medicine, The University of Hong Kong, Hong Kong Special Administrative Region, China

**Keywords:** epidemiology, longitudinal study, mental illness, refugees, social participation

## Abstract

**Aims:**

By the end of 2022, an estimated 108.4 million individuals worldwide experienced forced displacement. Identifying modifiable factors associated with the mental illness of refugees is crucial for promoting successful integration and developing effective health policies. This study aims to examine the associations between the changes in the diversity of social participation and psychological distress among refugees throughout the resettlement process, specifically focusing on gender differences.

**Methods:**

Utilizing data from three waves of a longitudinal, nationally representative cohort study conducted in Australia, this study involved 2399 refugees interviewed during Wave 1, 1894 individuals interviewed during Wave 3 and 1881 respondents during Wave 5. At each wave, we assessed psychological distress and 10 types of social participation across 3 distinct dimensions, including social activities, employment and education. The primary analysis employed mixed linear models and time-varying Cox models. Gender-stratified analyses and sensitivity analyses were performed.

**Results:**

Refugees engaging in one type or two or more types of social participation, compared with those not engaging in any, consistently had lower psychological distress scores (*β* = −0.62 [95% confidence interval (CI), −1.07 to −0.17] for one type of social participation; *β* = −0.57 [95% CI, −1.04 to −0.10] for two or more types of social participation) and a reduced risk of experiencing psychological distress (hazard ratio [HR] = 0.81 [95% CI, 0.65–0.99] for one type of social participation; HR = 0.77 [95% CI, 0.61–0.97] for two or more types of social participation) during the resettlement period. When stratifying the results by gender, these associations in the adjusted models only remained significant in male refugees. Moreover, three specific types of social participation, namely sporting activities, leisure activities and current employment status, were most prominently associated with a reduced risk of psychological distress.

**Conclusions:**

The findings of this cohort study suggest that social participation was consistently associated with reduced risks of psychological distress among male refugees during resettlement. These findings highlight the significance of promoting meaningful social participation and interaction may be an effective strategy to improve the mental health of refugees and facilitate their successful integration into society, especially among male refugees.

## Introduction

By the end of 2022, an estimated 108.4 million individuals were forcibly displaced globally, marking the highest number ever recorded. Among them, 40.7 million were refugees and asylum seekers (Agency, 2023). This growing exodus of refugees from their home countries is primarily driven by factors such as war, civil unrest, political violence and other humanitarian crises (Saadi *et al.*, 2021). Throughout their migration journey, refugees often endure cumulative trauma, including exposure to violence, human trafficking, unemployment, loneliness and challenges related to social integration (Chen *et al.*, 2017; Steel *et al.*, 2009). Consequently, the prevalence of mental illness within this population is notably high (Chen *et al.*, 2017; Saadi *et al.*, 2021). Although estimates indicate that the majority of global migration occurs within low-income and middle-income countries (LMICs), there has also been a substantial increase in migration towards high-income countries (HICs) (Abubakar *et al.*, 2018; Matlin *et al.*, 2018). Existing evidence suggests that refugees resettled in both LMICs and HICs exhibit a heightened frequency of psychiatric disorders compared with the general population (Barbui *et al.*, 2022; Peconga and Høgh Thøgersen, 2019). Identifying modifiable factors associated with mental illness is of paramount importance in planning public health interventions and promoting the overall well-being of refugees.

Social participation encompasses various activities, such as engaging in social groups, volunteering and employment, that facilitate interaction with others in society or the community (Levasseur *et al.*, 2010). Engaging in diverse activities offers opportunities to build social relationships and share emotional support, ultimately reducing feelings of loneliness and lowering the risk of psychological distress (Li *et al.*, 2018; Park *et al.*, 2013). Although previous studies have explored the association between social participation and mental illness, most of these studies have primarily focused on general older adults. For example, recent cross-sectional studies have demonstrated an association between social participation and a decreased risk of depressive symptoms among older adults (Choi *et al.*, 2021; Sun and Lyu, 2020). Additionally, longitudinal studies conducted in Australia and China have revealed that frequent social participation predicts better mental health outcomes in older adults (Ding *et al.*, 2015; Liu *et al.*, 2022). The heightened risk of isolation among general older adults is often attributed to the natural shrinking of their social networks with age (Blazer, 2005). Similarly, refugees face vulnerability to social isolation as they integrate into their host countries, which can lead to feelings of marginalization and reduced access to social support (Makhoul and Nakkash, 2009). Consequently, facilitating opportunities for employment, education and meaningful social engagement has been shown to alleviate mental health symptoms in refugees and other marginalized populations (Saadi *et al.*, 2021). For instance, previous studies have indicated that community involvement and employment prospects can enhance psychological well-being among refugees resettled in Bangladesh and Jordan (Hossain *et al.*, 2021; Wells *et al.*, 2016). Our recent study similarly revealed a negative association between paid employment and mental illness among refugees residing in Australia (Jiang *et al.*, 2023). Drawing upon the role accumulation theory, participating in various social activities may have beneficial compensatory or complementary effects (Adelmann, 1994; Choi *et al.*, 2021). However, few studies have explored the combined effects of engaging in different types of social participation on mental health, especially among refugees. Furthermore, most previous studies have employed cross-sectional designs for the general population or prospective designs that considered only social participation at baseline, neglecting changes in social participation over time. As a result, there is a notable absence of longitudinal studies investigating changes in the diversity of social participation and its impact on psychological distress.

Gender is a crucial organizing principle that underlies migration and related processes, influencing both who migrates and how these migrations occur (Monica Boyd, 2003). It also plays a significant role in shaping the experiences and futures of migrant women and families (Monica Boyd, 2003). Existing evidence among the general population suggests that older women may derive more significant mental health benefits from social participation than men (Choi *et al.*, 2021; Takagi *et al.*, 2013). Therefore, it is essential to explore potential gender differences in the association between the diversity of social participation and mental illness among refugees during resettlement, an area that has received limited attention.

To address these research gaps, the present study aims to examine the dynamic associations between the diversity of social participation and psychological distress among refugees through the resettlement period, taking into account multidimensional social participation, which includes various social activities, employment and education. Moreover, we explore the potential existence of gender differences in the association between social participation and mental illness.

## Methods

### Study design and population

The Building a New Life in Australia (BNLA) project is a nationwide survey focused on refugees, aimed at tracking the settlement experiences of recently resettled refugees in Australia and examining the associated outcomes and risk factors (Edwards *et al.*, 2018). Participants in the BNLA project come from diverse backgrounds and have followed various migration paths, seeking refuge in Australia to escape the ravages of war, civil unrest, political turmoil and other humanitarian crises (Edwards *et al.*, 2018; Saadi *et al.*, 2021). During the early resettlement stage (Wave 1) of the BNLA, it was observed that approximately 66.4% of refugees had limited proficiency in spoken English (Dowling *et al.*, 2019). This longitudinal study utilized data collected during three distinct waves of the BNLA project, spanning from October 2013 to February 2018, identified explicitly as Wave 1, Wave 3 and Wave 5. During these waves, the survey employed computer-assisted self-interviews (CASIs) conducted on portable computer tablets, including information on social participation (Rioseco *et al.*, 2017). CASI allows participants to privately respond to self-reported questions using a computer interface, potentially reducing reporting bias (Edwards *et al.*, 2018). For those who preferred an interview, a computer-assisted personal interview was also available. Moreover, to accommodate the diverse cultural and linguistic backgrounds of individuals in the study, survey materials were offered in English and 14 other translated languages (including Amharic, Arabic, Burmese/Myanmar, Chin Haka, Dari, Hazaragi, Nepali, Oromo, Pashto, Persian, Somali, Swahili, Tamil and Tigrinya) (Rioseco *et al.*, 2017). For languages beyond the translated languages, participants could complete questionnaires through interpreter-assisted interviews with trained interpreters. Including interpreters, a total of 19 languages were used in the BNLA project (Edwards *et al.*, 2018; Wu *et al.*, 2021). For this study, 2399 participants completed the interview in Wave 1. During the follow-up period, 1894 participants completed the interview in Wave 3, yielding a retention rate of 78.9%, and 1881 participants completed the interview in Wave 5, with a retention rate of 78.4% (Department of Social Services & Australian Institute of Family Studies, 2022). A detailed description of the participant selection process is provided in the eMethods and eFigure 1 in the Supplement.

### Measurement of psychological distress

The Kessler Screening Scale for Psychological Distress (K6) was utilized to measure nonspecific psychological distress at each wave, which includes two subscales (i.e., K6-Depression and K6-Anxiety). Each subscale consists of six items designed to evaluate the presence of symptoms such as nervousness, negative affect, fatigue, irritability, worthlessness and hopelessness experienced within the preceding 4 weeks (Lace *et al.*, 2020). The Australian version of K6 utilizes a 5-point rating scale, ranging from 1 (none of the time) to 5 (all of the time), yielding a total score between 6 and 30, with higher scores indicating more severe psychological distress.

In accordance with the cutoff point recommended by the Australian Bureau of Statistics, respondents scoring 19 or higher were categorized as experiencing psychological distress (Department of Social Services & Australian Institute of Family Studies, 2022). This measurement tool has been employed across diverse cultural contexts and has exhibited favourable psychometric properties (Hajebi *et al.*, 2018). Additionally, it has shown concurrent validity (Lace *et al.*, 2020). In the BNLA study, the internal consistency of the K6 remained consistently high across all five waves, with Cronbach’s *α* ranging from 0.87 to 0.92 (Garton *et al.*, 2022).

### Diversity of social participation

Information regarding social participation was collected during the Wave 1, Wave 3 and Wave 5 surveys. Based on previous studies (Adamakis, 2022; Choi *et al.*, 2021; Guo *et al.*, 2018), we identified 10 distinct indicators of social participation spanning 3 primary domains: social activity (i.e., sporting activities, leisure activities, school involvement, parent support groups, volunteering activities, cultural activities and self-improvement activities), employment (i.e., current paid employment in any job, business or on a farm) and education (i.e., current English language study and current job training or other studies). Each indicator of social participation was dichotomized and coded as either 0 or 1, indicating the absence or presence of the corresponding aspect of participation, respectively. For social activities, involvement on a daily/weekly/monthly basis was scored as 1, while participation a few times a year or less/never was scored as 0. For employment, having held paid employment in the past year was scored as 1, and 0 otherwise. For education, currently pursuing studies was scored as 1, and 0 otherwise. Detailed definitions and coding for each specific indicator of social participation can be found in eTable 1 in the Supplement. To further explore the impact of the diversity of social participation on psychological distress, we calculated social participation scores ranging from 0 to 10. Subsequently, participants were categorized into three groups based on the cumulative scores of the three dimensions of social participation: no social participation (score of 0), engagement in one type of social participation (score of 1) and engagement in two or more types of social participation (score of 2 and higher) (Choi *et al.*, 2021).

### Other variables

In this study, gender was recorded. Moreover, covariates were selected following a thorough review of data from the BNLA database and previous literature (Department of Social Services & Australian Institute of Family Studies, 2022; Sun and Lyu, 2020; Wu *et al.*, 2021). The covariates under consideration encompassed self-reported age, country of origin, marital status, educational level and weekly income at each wave. Country of origin was determined based on the information provided in participants’ visa applications, specifically their country of birth. Educational level was defined as the highest level of education completed prior to immigrating to Australia. Weekly income for participants included both self-reported salaries and government benefits and allowances.

### Statistical analysis

Baseline participant characteristics were summarized as mean (SD) or median (IQR) for continuous variables and as number and percentage for categorical variables. First, we presented baseline characteristics stratified by gender and different levels of social participation. Additionally, we described the prevalence of psychological distress and the patterns of social participation over three waves for both genders.

Second, given the changes in the diversity of social participation and psychological distress during resettlement, data from all three waves were included in our analyses. To assess the longitudinal association between the diversity of social participation and psychological distress scores (K6 scores) during resettlement, we employed mixed linear models with individuals treated as random effects (Xu *et al.*, 2023). The use of mixed linear models allowed us to leverage the information available in cohort studies and assess dynamic associations between the diversity of social participation and psychological distress scores over time (Finucane *et al.*, 2007). Moreover, to better understand the dynamic associations between the diversity of social participation and the presence of psychological distress, we utilized weighted time-varying Cox regression, using follow-up time as the timescale (Zhang *et al.*, 2018), to calculate hazard ratios (HRs) along with their corresponding 95% confidence intervals (CIs). These Cox models incorporated a time-varying covariate to account for changes in social participation over the follow-up period (Xu *et al.*, 2023). This approach is beneficial when exploring the associations between factors such as social participation and outcomes such as psychological distress, which are likely to change dynamically over time and are likely to be influenced by other time-varying covariates (Xu *et al.*, 2023). Longitudinal weights were applied to adjust attrition estimates in the follow-up survey to match the baseline recruited sample (Wu *et al.*, 2021). Initially, crude models were constructed, and then adjusted models were developed, controlling for age, country of origin, marital status, educational level and weekly income.

Moreover, several additional analyses were conducted. First, we conducted a series of univariable Cox regression models to evaluate the associations between each covariate and the presence of psychological distress. Second, we recategorized social participation as a dichotomous variable (i.e., no social participation and engagement in at least one type of social participation), and we included an interaction term of social participation × gender in the weighted time-varying Cox regression to assess the interactions between social participation and gender (Golaszewski *et al.*, 2022). Third, we also examined the interaction between social participation and country of origin for psychological distress by including the country of origin as a dummy or dichotomous variable (Golaszewski *et al.*, 2022; Lindley and Walker, 1993). Fourth, we employed weighted time-varying Cox regression models, separately incorporating each adjusting covariate, to identify covariates that could potentially confound the associations between social participation and psychological distress among female refugees. Fifth, we employed mixed linear models and time-varying Cox regression to explore the association between each type of social participation and psychological distress.

To test the robustness of our findings, we performed sensitivity analysis by using the multiple imputation by chained equations method to impute missing data (20 imputed datasets) and repeating mixed linear models with imputed datasets. Additionally, we conducted time-varying Cox regression models based on unweighted data. All analyses were conducted using Stata 17.0 (Stata Corporation, College Station, TX, USA), with two-tailed *P* < 0.05 considered statistically significant.

## Results

### Characteristics of the study participants

**Figure 1.**
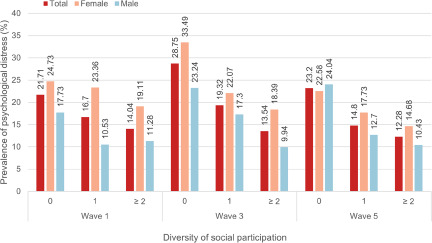
Prevalence of psychological distress in each wave among male and female refugees, based on the diversity of social participation.

Among the 2399 participants included in Wave 1, 1307 (54.5%) were male, with a mean age of 35.47 (SD: 13.91) years. Compared with their male counterparts, female refugees were more likely to report no social participation (187 of 1307 [20.66%] vs 144 of 1092 [12.97%]) (Table 1). Additionally, they exhibited less involvement in sporting activities, leisure activities and employment opportunities (eTable 2 in the Supplement). Participants who reported no social participation tended to be older and have lower levels of education (eTable 3 in the Supplement). As demonstrated in Fig. 1, for both genders, refugees who reported no social participation showed a higher prevalence rate of experiencing psychological distress when compared with those engaging in either one type or two or more types of social participation in each wave. For instance, among female refugees in Wave 1, the prevalence rate was 24.73% for those reporting no social participation, as opposed to 19.11% for those engaging in two or more types of social participation.

**Table 1.** Characteristics of participants at baseline stratified by gender

	*N* (%)^a^		
Variable	Total (*n* = 2399)	Male (*n* = 1307)	Female (*n* = 1092)
**Age at baseline, mean (SD), year**	35.47 (13.91)	35.69 (13.82)	35.21 (14.01)
**Country of origin**			
North Africa and the Middle East	1334 (55.61)	692 (52.94)	642 (58.79)
Southern and Central Asia	829 (34.56)	499 (38.18)	330 (30.22)
Southeast and Northeast Asia	139 (5.79)	69 (5.28)	70 (6.41)
Others	97 (4.04)	47 (3.60)	50 (4.58)
**Marital status**			
Married	1326 (57.60)	789 (62.57)	537 (51.58)
Never married	748 (32.49)	450 (35.69)	298 (28.63)
Separated/divorced/widowed	228 (9.90)	22 (1.74)	206 (19.79)
**Educational level**			
Never attended school	380 (15.99)	167 (12.94)	213 (19.63)
≤6 years of schooling	473 (19.91)	266 (20.60)	207 (19.08)
>6 years of schooling	1137 (47.85)	627 (48.57)	510 (47.00)
Trade or technical qualification, or university degree	386 (16.25)	231 (17.89)	155 (14.29)
**Weekly income, median (IQR), AU$**	230 (204, 280)	230 (210, 270)	240 (200, 295)
**Diversity of social participation**			
0	331 (16.43)	144 (12.97)	187 (20.66)
1	965 (47.89)	501 (45.14)	464 (51.27)
≥2	719 (35.68)	465 (41.89)	254 (28.07)

Abbreviations: SD, standard deviation; IQR, interquartile range.

a Data were shown as mean (SD) for continuous variables (age) and number with proportion (*n* [%]) for categorical variables.

### Association between the diversity of social participation and psychological distress scores during resettlement

As shown in Table 2, in mixed linear models, engaging in one type and two or more types of social participation exhibited a negative association with psychological distress scores through the resettlement process in both the crude and adjusted models (*β* = −0.62 [95% CI, −1.07 to −0.17] for one type of social participation; *β* = −0.57 [95% CI, −1.04 to −0.10] for two or more types of social participation in the adjusted models). Stratification analyses showed significant negative associations of engaging in one type and two or more types of social participation with psychological distress scores among male refugees, with and without adjusting for covariates (e.g., *β* = −0.66 [95% CI, −1.29 to −0.03] for one type of social participation; *β* = −0.70 [95% CI, −1.36 to −0.05] for two or more types of social participation in the adjusted models). However, among female refugees, a negative association between the diversity of social participation and psychological distress scores was observed in the crude model (*β* = −0.74 [95% CI, −1.30 to −0.17] for one type of social participation; *β* = −0.78 [95% CI, −1.39 to −0.18] for two or more types of social participation); nonetheless, this relationship did not attain statistical significance after adjusting for covariates.

**Table 2.** Mixed linear models for the longitudinal association of psychological distress scores with the diversity of social participation stratified by gender

	*β* (95% CI)^a^					
	Total		Male		Female	
	Crude model	Adjusted model	Crude model	Adjusted model	Crude model	Adjusted model
Diversity of social participation						
0	Reference	Reference	Reference	Reference	Reference	Reference
1	−1.07 (−1.47 to −0.67)	−0.62 (−1.07 to −0.17)	−1.27 (−1.84 to −0.69)	−0.66 (−1.29 to −0.03)	−0.74 (−1.30 to −0.17)	−0.52 (−1.17 to 0.14)
≥2	−1.31 (−1.73 to −0.90)	−0.57 (−1.04 to −0.10)	−1.57 (−2.15 to −0.99)	−0.70 (−1.36 to −0.05)	−0.78 (−1.39 to −0.18)	−0.27 (−0.97 to 0.44)

Abbreviations: *β*, regression coefficient; 95% CI, 95% confidence interval.

a Adjusted models were controlled for age, country of origin, marital status, educational level and weekly income.

### Associations between the diversity of social participation and the presence of psychological distress during resettlement

Table 3 presents associations between the diversity of social participation and the presence of psychological distress. In time-varying Cox regression analysis, when compared with individuals with no social participation, those engaged in either one type or two or more types of social participation demonstrated a reduced risk of experiencing psychological distress, as shown in the adjusted models (HR = 0.81 [95% CI, 0.65–0.99] for one type of social participation; HR = 0.77 [95% CI, 0.61–0.97] for two or more types of social participation). Furthermore, when stratifying the results by gender, it was observed that social participation had a positive impact on reducing the presence of psychological distress in male refugees, and this association remained significant even after adjusting for covariates (HR = 0.64 [95% CI, 0.45–0.89] for one type of social participation; HR = 0.68 [95% CI, 0.48–0.96] for two or more types of social participation). However, among females, engaging in two or more types of social participation was associated with a lower risk of having psychological distress in the crude model (HR = 0.72 [95% CI, 0.55–0.95]), but this association did not reach statistical significance in the adjusted model.

**Table 3.** Time-varying Cox regression models for weighted associations of experiencing psychological distress with the diversity of social participation stratified by gender^a^

	Hazard ratios of psychological distress (95% CI)^b^					
	Total		Male		Female	
	Crude model	Adjusted model	Crude model	Adjusted model	Crude model	Adjusted model
Diversity of social participation						
0	1.00 (reference)	1.00 (reference)	1.00 (reference)	1.00 (reference)	1.00 (reference)	1.00 (reference)
1	0.68 (0.56–0.84)	0.81 (0.65–0.99)	0.53 (0.39–0.73)	0.64 (0.45–0.89)	0.87 (0.68–1.12)	0.99 (0.75–1.31)
≥2	0.59 (0.48–0.72)	0.77 (0.61–0.97)	0.52 (0.38–0.71)	0.68 (0.48–0.96)	0.72 (0.55–0.95)	0.92 (0.67–1.26)

Abbreviation: 95% CI, 95% confidence interval.

a Time-varying Cox regression models were weighted using longitudinal weights.

b Adjusted models were controlled for age, country of origin, marital status, educational level and weekly income.

### Additional and sensitivity analyses

The univariable analysis of covariates showed that age, country of origin or marital status were significantly associated with psychological distress (eTable 4 in the Supplement). Moreover, we observed a significant multiplicative interaction between social participation and gender concerning psychological distress in crude and adjusted models (eTable 5 in the Supplement). Conversely, the interaction between social participation and country of origin associated with psychological distress was not statistically significant (eTables 6–7 in the Supplement). In models incorporating each covariate separately, we observed a significant association between social participation and psychological distress among female refugees, even after adjusting for educational level or weekly income. However, this significant association disappeared after adjusting for age, country of origin or marital status (eTable 8 in the Supplement). The adjusted models revealed three specific types of social participation, namely sporting activities, leisure activities and current employment status, were most prominently associated with a reduced risk of psychological distress (eTables 9–10 in the Supplement). Moreover, our sensitivity analysis showed that when we performed mixed linear models with imputed datasets and conducted time-varying Cox regression using unweighted data, the results consistently supported our main findings (eTables 11–12 in the Supplement).

## Discussion

In this national longitudinal study conducted among refugees, individuals who participated in at least one type of social participation exhibited lower psychological distress scores and a reduced risk of experiencing psychological distress during resettlement compared with those who did not engage in any social participation. Stratified analyses revealed that among male refugees, engaging in either one type or two or more types of social participation demonstrated a consistently negative association with both psychological distress scores and experiencing psychological distress, both in the crude and adjusted models. Conversely, the initially observed negative associations among female refugees were no longer statistically significant in the adjusted model.

In this 5-year longitudinal study, we observed that refugees who participated in at least one type of social participation had a lower risk of psychological distress than those who did not engage in any social participation. Furthermore, engaging in two or more types of social participation was associated with the lowest risk of having psychological distress. Previous studies have primarily focused on the general population when examining the association between social participation and mental health (Choi *et al.*, 2021; Mackenzie and Abdulrazaq, 2021; Sun and Lyu, 2020). For example, earlier cross-sectional studies conducted in Korea and China have shown that more frequent and diverse participation in activities is associated with reducing depressive symptoms among older adults in the general population (Choi *et al.*, 2021; Guo *et al.*, 2018). Notably, both refugees and older adults are susceptible to social isolation due to significant reductions in their social networks, which makes them vulnerable to mental illness (Blazer, 2005; Makhoul and Nakkash, 2009). However, there is limited research that examines the association between social participation and psychological distress, specifically in refugees through the resettlement process. For instance, a cross-sectional study conducted in Sweden demonstrated that social participation had a protective effect on the mental health of migrants, particularly when they were exposed to risk factors such as discrimination (Lecerof *et al.*, 2016). Another cross-sectional study revealed that social integration factors played a pivotal role in explaining the mental health disparities between native-born individuals and migrants in Sweden (Brydsten *et al.*, 2019). It is worth noting that the cross-sectional nature of these studies limits their ability to consider the changes in social participation or psychological health during follow-up. Our findings from the longitudinal data suggest that the diversity of social participation is consistently beneficial to refugees’ psychological distress during resettlement. Several hypotheses could explain the protective effect of the diversity of social participation on mental health. First, according to the stress-buffering hypothesis, positive social interactions can help mitigate psychological distress stemming from cumulative trauma (Cohen and Wills, 1985). Second, refugees may face social exclusion and language barriers that hinder their social integration after arrival in host countries, and engaging in social participation can assist refugees in achieving social inclusion and better integration into the host country (Betancourt *et al.*, 2015; Mangrio *et al.*, 2022). Third, from the perspective of role accumulation theory, engaging in multiple roles through diverse social activities may have beneficial compensatory or complementary effects (Adelmann, 1994; Choi *et al.*, 2021). Increased social participation might provide refugees better access to resources and support, improving culture integration, self-efficacy and a sense of belonging, thereby reducing psychological distress. Therefore, promoting individual engagement in diverse and meaningful social participation merits elevated attention in interventions aimed at improving the mental health of refugees.

Additionally, although our stratification analyses demonstrated a significantly negative association between the diversity of social participation and psychological distress in both male and female refugees in the unadjusted models, in the models adjusting for all covariates (including age, country of origin, marital status, educational level and weekly income), only the association among male refugees remained significant. While prior research has suggested that gender plays a vital role in understanding the impact of social participation and its consequences (Agahi and Parker, 2008), only a limited number of studies have explicitly examined the relative importance of social participation for male and female refugees individually (Khawaja *et al.*, 2006). In this study, male refugees reported a higher degree of social participation than their female counterparts, which aligns with previous findings indicating elevated levels of social participation among older males (Choi *et al.*, 2021). This finding can be contextualized within the migration framework suggested by our previous research, where male refugees often have increased opportunities to engage in paid employment and participate in various activities compared with female refugees (Jiang *et al.*, 2023). The main argument of migration literature is that females often play a secondary role in family migration decisions, resulting in their lower human capital and earning potential in the destination country (Cooke *et al.*, 2009). Correspondingly, immigrant male networks are more strongly linked to work and include more individuals beyond kinship boundaries, while immigrant female social networks consist predominantly of family and relatives (Schrover *et al.*, 2007). Female refugees mainly stay in a family context and spend less time than males with nonimmigrants or people living in the host country for a longer period (Kosyakova and Kulic, 2022; Steinmann, 2021). Thus, they miss the opportunity to build a broad social network supporting them (Yuliya Kosyakova, 2023). Furthermore, previous evidence suggests that during resettlement and migration, female refugees often tend to prioritize their families and the future of their children (Paoletti *et al.*, 2023). Their self-efficacy appears to be strengthened through child-rearing rather than extensive social participation (Ahrens and Ryff, 2006). In contrast, male refugees may derive greater self-esteem and empowerment from diverse social participation, including employment, social activities and education (Jiang *et al.*, 2023). However, it is important to note that previous studies conducted among the general population in Japan and Korea have documented a more significant influence of social participation on self-reported mental health among older women than older men (Mackenzie and Abdulrazaq, 2021; Takagi *et al.*, 2013). The inconsistencies in the results might be attributed to the fact that most existing literature explores the influence of social participation among non-refugee populations, particularly among older adults, and often focuses on limited types of social participation. Further high-quality research is imperative to corroborate the gender differences in the association between social participation and mental illness in the future.

We further observed that the initially unadjusted significant association between social participation and psychological distress among female refugees became nonsignificant upon adjustment for age, country of origin or marital status. This finding suggests that age, country of origin and marital status may act as confounding variables that influence the association between social participation and psychological distress among female refugees. A potential explanation is that age might be a crucial factor in the association of social participation with mental health among females, with older women being more likely to derive substantial benefits from social activities (Matud *et al.*, 2015; Tomioka *et al.*, 2017). Our results were consistent with a previous study in the BNLA that showed a significant association between country of origin and refugees’ mental health (Chen *et al.*, 2017). Generally, the social participation of immigrant females corresponds to the female social participation in the country of origin (Blau and Kahn, 2015). In this sense, traditional family roles and care responsibilities can constrain female social attachment; such roles and responsibilities seem to be more pronounced among immigrants from predominantly Muslim countries (Khoudja and Fleischmann, 2015). Moreover, marital status could be another critical factor associated with refugees’ mental health and their extent of social participation, particularly for females. Our results showed that those who never married had a higher percentage of engaging in social participation and a lower risk of mental illness. Married female refugees are usually focused on looking after family and could be constrained by traditional social perceptions, limiting their social participation (Donato *et al.*, 2014; Yuliya Kosyakova, 2023).

Moreover, our results have demonstrated that among the various types of social participation considered in the study, sporting activities, leisure activities or current paid employment were three types significantly associated with a reduced risk of psychological distress among refugees. These findings are consistent with previous evidence suggesting that engaging in meaningful and purposeful daily activities, as well as being employed, can alleviate symptoms of mental illness in refugees (Chen *et al.*, 2017; Jiang *et al.*, 2023; Saadi *et al.*, 2021). Earlier studies have also demonstrated that physical activity and leisure pursuits can enhance interpersonal communication and bolster mental health resilience (Adamakis, 2022; Copeland *et al.*, 2022). According to the broaden-and-build theory, engaging in leisure activities that elicit positive emotions can augment mental resources for coping with stress (Takiguchi *et al.*, 2023). Furthermore, previous research has proposed that paid employment offers economic support and opportunities for social interaction, both of which are crucial for sustaining mental health (Hossain *et al.*, 2021). Therefore, providing refugees with employment opportunities and establishing a supportive and inclusive environment to enhance social participation could be the focus of intervention throughout the resettlement process to promote refugees’ psychosocial adjustment, especially among male refugees.

The strengths of this study include its prospective design, a national refugee-based sample, the inclusion of diverse social participation measures and an extended follow-up for psychological distress, allowing the assessment of social participation dynamic changes and providing insight into the nature of the association between the diversity of social participation and psychological distress. However, there are several limitations. First, although standardized and validated cultural and social context methods are used in data collection, recall and reporting bias in the questionnaire data cannot be ruled out entirely. Second, it should be noted that the K-6 scale is based on DSM-IV, as the project was initiated prior to the release of DSM-V. Third, although the study sample broadly represents refugees in Australia, the generalizability of our results to those residing in LMICs may be limited due to potential disparities in community and health services offered to refugees between HICs and LMICs (Abubakar *et al.*, 2018). Therefore, caution should be exercised when generalizing these findings to refugees in LMICs and non-refugee populations. Fourth, there remains the possibility of residual confounding due to unmeasured factors such as genetics that could potentially exert an influence on the estimated association. Fifth, due to the nature of observational studies, causal relationships between social participation and psychological distress cannot be fully established (Hammerton and Munafò, 2021), necessitating future randomized clinical trials to confirm our findings.

## Conclusion

Our findings suggest that engaging in at least one type of social participation is consistently associated with a reduced risk of psychological distress among refugees during resettlement, with a particularly notable effect observed among male refugees. This study highlights the significance of social participation, especially in sporting activities, leisure activities and paid employment, as protective factors for mental health in refugees. Policymakers are suggested to prioritize promoting such engagement and addressing barriers to participation through the resettlement period to enhance the mental well-being of refugees and facilitate their successful integration into society.

## Supporting information

Jiang et al. supplementary material Jiang et al. supplementary material

## Data Availability

All relevant data can be found in the following repository: https://bnla.aifs.gov.au/. The analysis code for this study can be obtained upon request from the corresponding author.

## Supplementary material

The supplementary material for this article can be found at https://doi.org/10.1017/S2045796024000313.
