# Supplementary material for: Contribution of diversity of social participation on the mental health of humanitarian migrants during resettlement
Source: Epidemiol Psychiatr Sci. 2024 May 23;33:e29. doi: 10.1017/S2045796024000313 (PMC11362679; doi:10.1017/S2045796024000313)
Supplement: Jiang et al. supplementary material [file S2045796024000313sup001.docx]

**Contribution of diversity of social participation on the mental health of humanitarian migrants during resettlement**

eMethods. Study Design and Population

eFigure 1. Flowchart Illustrating the Selection of Study Participants

eTable 1. Questionnaire Items for Each Type of Social Participation

eTable 2. Overview of Social Participation Involvement Across Three Waves, Stratified by Gender

eTable 3. Baseline Participant Characteristics Stratified by Diversity of Social Participation

eTable 4. Univariable Time-varying Cox Regression Models for Weighted Associations of Each Covariate with Psychological Distress

eTable 5. Interaction Analysis of Social Participation and Gender on Psychological Distress

eTable 6. Interaction Analysis of Social Participation and Country of Origin (Dummy Variable) on Psychological Distress

eTable 7. Interaction Analysis of Social Participation and Country of Origin (Dichotomous Variable) on Psychological Distress

eTable 8. Time-varying Cox Regression Models for Weighted Associations of Social Participation Diversity with Psychological Distress, Adjusted for Each Covariate Separately

eTable 9. Longitudinal Mixed Linear Models Examining the Association of Different Types of Social Participation with Psychological Distress Scores, Stratified by Gender

eTable 10. Time-varying Cox Regression Models for Weighted Associations of Different Types of Social Participation with Psychological Distress, Stratified by Gender

eTable 11. Longitudinal Mixed Linear Models Assessing the Association of Social Participation Diversity with Psychological Distress Scores, Stratified by Gender, Using Imputed Datasets.

eTable 12. Time-varying Cox Regression Models for Unweighted Associations of Social Participation Diversity with Psychological Distress, Stratified by Gender

**eMethods. Study Design and Population**

The BNLA study is a national refugee-based survey that used a prospective cohort design to trace the settlement journey of recently resettled refugees in Australia and to investigate outcomes and risk factors related to this process (Edwards *et al.*, 2018). The baseline survey, also known as Wave 1, was conducted between October 1, 2013, and February 29, 2014. The follow-up surveys, Wave 3 and Wave 5, were administered between October 1, 2015, and February 29, 2016, and between October 1, 2017 and February 29, 2018, respectively (Department of Social Services & Australian Institute of Family Studies, 2022). First, 11 cities with the most significant number of refugees in Australia from November 2010 to October 2011 were selected. Second, the researchers obtained the list of all eligible migrants from the Australian Government’s Department of Immigration and Border Protection (n=4035), who were aged 18 or older, were the principal applicants for permanent humanitarian migrant visas, and had lived in Australia for 3–6 months before the first wave of the survey. Finally, of the 2031 refugees successfully contacted after the initial contact, 1509 agreed to participate in the project. Their family members aged 15 years or older and in the same visa application were also invited to participate in the project (n=890). These refugees (n=2399) participated in the baseline survey (Wave 1) from October 2013 to March 2014 and were followed up annually between October and February of the following year. In the following waves, an initial contact through a Primary Approach Letter (PAL) was sent to each participant who had completed the Wave 1 interview and had not subsequently withdrawn from the project. Through the PAL, participants were reminded that field researchers would contact them by telephone to arrange appointments to complete interviews. To date, four follow-up visits have been completed. Data were collected using computer-assisted self-interviews (CASI) or computer-assisted personal interviews (CAPI) in Waves 1, 3, and 5 and telephone interviews in Waves 2 and 4 (Rioseco *et al.*, 2017).

The present longitudinal study utilized data from three waves of the BNLA project (i.e., Wave 1, Wave 3, and Wave 5, 2013-2018), which collected data through interviews and contained information on social participation. CASI enables participants to privately respond to self-reported questions using a computer interface, which may reduce reporting bias (Edwards *et al.*, 2018). To accommodate the diverse cultural and linguistic backgrounds of individuals in the study, the survey materials were offered in English or other translated 14 languages (Rioseco *et al.*, 2017). Moreover, for languages beyond the translated languages, participants were able to complete questionnaires through interpreter-assisted interviews with trained interpreters. Including interpreters, a total of 19 languages were used in the BNLA project (Edwards *et al.*, 2018; Wu *et al.*, 2021). The study included all refugees who completed questionnaires at each wave. A total of 2399 refugees were in Wave 1. During follow-up, a total of 1894 in Wave 3 (retention rate = 78.9%) and 1881 in Wave 5 (retention rate = 78.4%) completed the interview. The data guide of the BNLA study showed that the study had a lower representation of refugees who lived in capital cities in Australia, smaller-sized families with a non-unauthorized maritime arrival visa subclass, and were born in Burma, the rest of Asia or Africa (Department of Social Services & Australian Institute of Family Studies, 2022). Therefore, in our analyses, survey weights provided in BNLA datasets were used to adjust the representativeness of the sample.

**
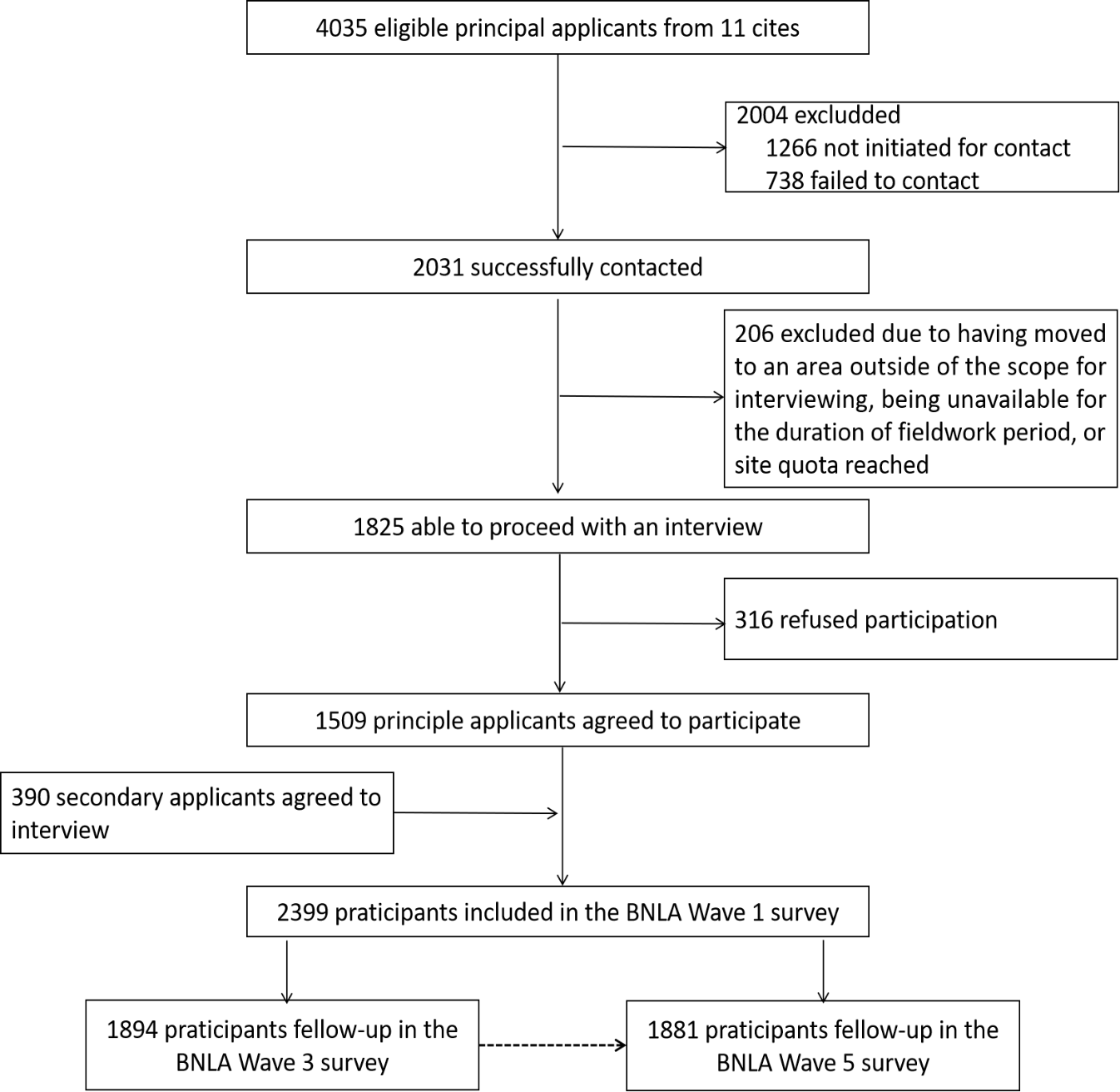
**

**eFigure 1. Flowchart Illustrating the Selection of Study Participants**

| **eTable 1. Questionnaire Items for Each Type of Social Participation** | | |
| --- | --- | --- |
| **Social participation indicators** | **Questionnaire items** | **Coding** |
| **Social activities** | | |
| Sporting activities | How often have you been involved in Sporting activities? | 0=A few times a year or less/Never 1=Daily/Weekly/Monthly |
| Leisure activities | How often have you been involved in leisure activities (e.g. movie nights, cooking classes)? |  |
| School involvement | How often have you been involved in school activities? |  |
| Parent support groups | How often have you been involved in parent support groups? |  |
| Volunteering activities | How often have you been involved in volunteering/helping others in your ethnic/religious community? |  |
| Cultural activities | How often have you been involved in cultural activities (e.g. festivals, special days)? |  |
| Self-improvement activities | How often have you been involved in self-improvement activities (e.g. coping with stress, exercise class)? |  |
| **Employment** | | |
| Current paid employment status | In the last 7 days did you do any paid work in a job, business, or on a farm? | 0=No  1=Yes |
| **Education** | | |
| Current English language study | Have you studied English since arriving in Australia? | 0=No/Yes, but I am no longer studying  1=Yes, I am currently studying |
| Current job training or other studies | Other than English language classes, have you undertaken any study or job training since you arrived in Australia? |  |

| **eTable 2. Overview of Social Participation Involvement Across Three Waves, Stratified by Gender** | | | | | | | | | |
| --- | --- | --- | --- | --- | --- | --- | --- | --- | --- |
| **Social participation indicators** | **Wave 1, n (%)** | | | **Wave 3, n (%)** | | | **Wave 5, n (%)** | | |
|  | **Total** | **Male** | **Female** | **Total** | **Male** | **Female** | **Total** | **Male** | **Female** |
| Sporting activities | 457 (20.92) | 310 (25.85) | 147 (14.91) | 378 (21.06) | 227 (23.45) | 151 (18.26) | 369 (20.31) | 210 (21.92) | 159 (18.51) |
| Leisure activities | 286 (13.10) | 182 (15.24) | 104 (10.52) | 283 (15.85) | 176 (18.33) | 107 (12.97) | 310 (17.15) | 179 (18.80) | 131 (15.30) |
| School involvement | 276 (12.31) | 128 (10.28) | 148 (14.84) | 255 (13.98) | 114 (11.69) | 141 (16.61) | 243 (13.19) | 120 (12.27) | 123 (14.24) |
| Parent support groups | 104 (4.64) | 53 (4.27) | 51 (5.09) | 103 (5.73) | 44 (4.57) | 59 (7.08) | 107 (5.83) | 61 (6.26) | 46 (5.34) |
| Volunteering activities | 188 (8.50) | 110 (9.10) | 78 (7.78) | 171 (9.41) | 98 (10.02) | 73 (8.69) | 184 (10.08) | 104 (10.87) | 80 (9.21) |
| Cultural activities | 234 (10.51) | 132 (10.85) | 102 (10.11) | 168 (9.16) | 91 (9.27) | 77 (9.04) | 131 (7.10) | 71 (7.33) | 60 (6.84) |
| Self-improvement activities | 84 (3.69) | 38 (3.03) | 46 (4.49) | 71 (3.88) | 30 (3.07) | 41 (4.81) | 158 (8.62) | 91 (9.46) | 67 (7.70) |
| Current paid employment status | 209 (8.78) | 191 (14.74) | 18 (1.66) | 516 (27.51) | 411 (41.10) | 105 (11.99) | 691 (36.97) | 537 (54.35) | 154 (17.48) |
| Current English language study | 1714 (72.50) | 945 (73.26) | 769 (71.60) | 760 (42.34) | 363 (38.09) | 397 (47.15) | 416 (22.89) | 161 (16.84) | 255 (29.62) |
| Current job training or other studies | 278 (11.73) | 152 (11.77) | 126 (11.69) | 397 (21.81) | 191 (19.61) | 206 (24.35) | 341 (18.53) | 150 (15.32) | 191 (22.18) |

| **eTable 3. Baseline Participant Characteristics Stratified by Diversity of Social Participation** ^a^ | | | |
| --- | --- | --- | --- |
| **Variable** | **No. of social participation indicators, n (%) ^b^** | | |
|  | **0 (n=331)** | **1 (n=965)** | **≥2 (n=719)** |
| Gender |  |  |  |
| Male | 144 (43.50) | 501 (51.92) | 465 (64.67) |
| Female | 187 (56.50) | 464 (48.08) | 254 (35.33) |
| Age at baseline, mean (SD), year | 44.36 (16.21) | 34.84 (13.33) | 32.45 (11.65) |
| Country of origin |  |  |  |
| North Africa and the Middle East | 244 (73.72) | 570 (59.07) | 351 (48.82) |
| Southern and Central Asia | 71 (21.45) | 334 (34.61) | 281 (39.08) |
| Southeast and Northeast Asia | 8 (2.42) | 30 (3.11) | 50 (6.95) |
| Others | 8 (2.42) | 31 (3.21) | 37 (5.15) |
| Marital status |  |  |  |
| Married | 249 (76.15) | 498 (53.72) | 373 (54.37) |
| Never married | 33 (10.09) | 327 (35.28) | 267 (38.92) |
| Separated/divorced/widowed | 45 (13.76) | 102 (11.00) | 46 (6.71) |
| Educational level |  |  |  |
| Never attended school | 73 (22.12) | 147 (15.26) | 93 (13.01) |
| ≤ 6 years of schooling | 82 (24.85) | 199 (20.66) | 126 (17.62) |
| > 6 years of schooling | 126 (38.18) | 461 (47.87) | 373 (52.17) |
| Trade or technical qualification, or university degree | 49 (14.85) | 156 (16.20) | 123 (17.20) |
| Weekly income, median (IQR), AU$ | 250 (228.0, 340.0) | 230 (214.5, 266.5) | 230 (135.0, 278.0) |

Abbreviation: SD: Standard Deviation; IQR: Inter-Quartile Range.

^a^ There were 384 participants with missing information on the number of social participation indicators at baseline.

^b^ Data were shown as mean (SD) for continuous variables (age) and number with proportion (n [%]) for categorical variables.

| **eTable 4. Univariable Time-varying Cox Regression Models for Weighted Associations of Each Covariate with Psychological Distress** ^a^ | | |
| --- | --- | --- |
| **Variable** | **Hazard ratios (95% CI)** | ***P* value** |
| Age | 1.02 (1.01 to 1.03) | <0.001 |
| Country of origin (ref= North Africa and the Middle East) |  |  |
| Southern and Central Asia | 0.57 (0.48 to 0.68) | <0.001 |
| Southeast and Northeast Asia | 0.20 (0.11 to 0.37) | <0.001 |
| Others | 0.40 (0.22 to 0.74) | 0.003 |
| Marital status (ref=married) |  |  |
| Never married | 0.80 (0.67 to 0.96) | 0.017 |
| Separated/divorced/widowed | 1.94 (1.61 to 2.33) | <0.001 |
| Educational level (ref= never attended school) |  |  |
| ≤ 6 years of schooling | 0.88 (0.69 to 1.12) | 0.297 |
| > 6 years of schooling | 0.96 (0.78 to 1.18) | 0.687 |
| Trade or technical qualification, or university degree | 1.07 (0.82 to 1.38) | 0.625 |
| Weekly income, AU$ | 0.99 (0.99 to 1.00) | 0.791 |

Abbreviations: 95% CI, 95% Confidence Interval.

^a^ Time-varying Cox regression models were weighted using longitudinal weights.

| **eTable 5. Interaction Analysis of Social Participation and Gender on Psychological Distress** ^a^ | | | | |
| --- | --- | --- | --- | --- |
|  | **Crude model** | | **Adjusted model ^c^** | |
|  | **Hazard ratios (95% CI)** | ***P* value** | **Hazard ratios (95% CI)** | ***P* value** |
| Social participation (ref=no) ^b^ | 0.52 (0.40 to 0.68) | <0.001 | 0.65 (0.48 to 0.86) | 0.003 |
| Gender (ref=male) | 1.11 (0.83 to 1.48) | 0.486 | 1.00 (0.73 to 1.37) | 0.990 |
| Social participation × Gender | 1.57 (1.10 to 2.22) | 0.012 | 1.50 (1.04 to 2.16) | 0.030 |

Abbreviations: 95% CI, 95% Confidence Interval.

^a^ Time-varying Cox regression models were weighted using longitudinal weights.

^b^ Social participation was recategorized as a dichotomous variable (i.e., 0 social participation indicator and 1 and higher social participation indicator).

^c^ Adjusted models were controlled for age, country of origin, marital status, educational level, and weekly income.

| **eTable 6. Interaction Analysis of Social Participation and Country of Origin (Dummy Variable) on Psychological Distress** ^a^ | | | | |
| --- | --- | --- | --- | --- |
|  | **Crude model** | | **Adjusted model ^d^** | |
|  | **Hazard ratios (95% CI)** | ***P* value** | **Hazard ratios (95% CI)** | ***P* value** |
| Social participation (ref=no) ^b^ | 0.91 (0.80 to 1.04) | 0.181 | 0.97 (0.84 to 1.12) | 0.705 |
| Country of origin ^c^ | 0.62 (0.50 to 0.77) | <0.001 | 0.60 (0.46 to 0.76) | <0.001 |
| Social participation × country of origin | 1.01 (0.93 to 1.10) | 0.816 | 0.99 (0.91 to 1.09) | 0.863 |

Abbreviations: 95% CI, 95% Confidence Interval.

^a^ Time-varying Cox regression models were weighted using longitudinal weights.

^b^ Social participation was recategorized as a dichotomous variable (i.e., 0 social participation indicator and 1 and higher social participation indicator).

^c^ Country of origin was set as a dummy variable:1=North Africa and the Middle East; 2=Southern and Central Asia; 3=Southeast and Northeast Asia; 4=others.

^d^ Adjusted models were controlled for age, gender, marital status, educational level, and weekly income.

| **eTable 7. Interaction Analysis of Social Participation and Country of Origin (Dichotomous Variable) on Psychological Distress** ^a^ | | | | |
| --- | --- | --- | --- | --- |
|  | **Crude model** | | **Adjusted model ^d^** | |
|  | **Hazard ratios (95% CI)** | ***P* value** | **Hazard ratios (95% CI)** | ***P* value** |
| Social participation (ref=no) ^b^ | 0.69 (0.57 to 0.84) | <0.001 | 0.83 (0.67 to 1.04) | 0.105 |
| Country of origin (ref=North Africa and the Middle East) ^c^ | 0.52 (0.35 to 0.78) | 0.001 | 0.51 (0.33 to 0.79) | 0.003 |
| Social participation × country of origin | 1.10 (0.70 to 1.72) | 0.692 | 0.94 (0.58 to 1.52) | 0.810 |

Abbreviations: 95% CI, 95% Confidence Interval.

^a^ Time-varying Cox regression models were weighted using longitudinal weights.

^b^ Social participation was recategorized as a dichotomous variable (i.e., 0 social participation indicator and 1 and higher social participation indicator).

^c^ Country of origin was recategorized as a dichotomous variable: North Africa and the Middle East, and Others.

^d^ Adjusted models were controlled for age, gender, marital status, educational level, and weekly income.

| **eTable 8. Time-varying Cox Regression Models for Weighted Associations of Social Participation Diversity with Psychological Distress, Adjusted for Each Covariate Separately ^a^** | | | | | | | | | | |
| --- | --- | --- | --- | --- | --- | --- | --- | --- | --- | --- |
|  | **Hazard ratios (95% CI)** | | | | | | | | | |
|  | **Male** | | | | | **Female** | | | | |
|  | **Model 1** | **Model 2** | **Model 3** | **Model 4** | **Model 5** | **Model 1** | **Model 2** | **Model 3** | **Model 4** | **Model 5** |
| Diversity of social participation |  | | | | | | | | | |
| 0 | Reference | | | | | Reference | | | | |
| 1 | 0.62 (0.44 to 0.86) | 0.61 (0.44 to 0.84) | 0.54 (0.39 to 0.73) | 0.53 (0.39, 0.72) | 0.51 (0.37, 0.70) | 1.00 (0.77 to 1.30) | 0.90 (0.70 to 1.16) | 0.94 (0.73 to 1.21) | 0.88 (0.68, 1.13) | 0.83 (0.63, 1.08) |
| ≥2 | 0.63 (0.45 to 0.89) | 0.62 (0.45 to 0.85) | 0.53 (0.39 to 0.72) | 0.50 (0.36, 0.68) | 0.52 (0.38, 0.70) | 0.88 (0.66 to 1.18) | 0.78 (0.59 to 1.02) | 0.79 (0.60 to 1.05) | 0.73 (0.55, 0.97) | 0.70 (0.52, 0.94) |

Abbreviations: 95% CI, 95% confidence Interval.

^a^ Time-varying Cox regression models were weighted using longitudinal weights.

Model 1 adjusted for age.

Model 2 adjusted for country of origin.

Model 3 adjusted for marital status.

Model 4 adjusted for educational level.

Model 5 adjusted for weekly income.

| **eTable 9. Longitudinal Mixed Linear Models Examining the Association of Different Types of Social Participation with Psychological Distress Scores, Stratified by Gender** | | | |
| --- | --- | --- | --- |
|  | **β (95% CI) ^a^** | | |
|  | **Total** | **Male** | **Female** |
| **Social participation types** |  |  |  |
| Sporting activities | -0.45 (-0.85 to -0.06) | -0.51(-1.00 to -0.01) | -0.19 (-0.84 to 0.46) |
| Leisure activities | -0.46 (-0.90 to -0.03) | -0.41 (-0.95 to 0.14) | -0.42 (-1.15 to 0.30) |
| School activities | -0.03 (-0.46 to 0.46) | 0.10 (-0.55 to 0.75) | -0.28 (-0.94 to 0.38) |
| Parent support groups | 0.41 (-0.28 to 1.10) | 0.52 (-0.42 to 1.46) | 0.14 (-0.89 to 1.16) |
| Youth groups | -0.03 (-0.68 to 0.61) | 0.01 (-0.84 to 0.85) | -0.03 (-1.03 to 0.98) |
| Volunteering activities | 0.32 (-0.21 to 0.86) | 0.30 (-0.38 to 0.97) | 0.38 (-0.49 to 1.25) |
| Cultural activities | 0.35 (-0.18 to 0.89) | 0.38 (-0.31 to 1.07) | 0.36 (-0.49 to 1.20) |
| Self-improvement activities | 0.58 (-0.00 to 1.16) | 0.70 (-0.03 to 1.43) | 0.47 (-0.48 to 1.43) |
| Current paid employment status | -1.13 (-1.51 to -0.74) | -0.89 (-1.34 to -0.44) | -0.94 (-1.75 to -0.14) |
| Current English study | 0.42 (0.10 to 0.73) | 0.31 (-0.10 to 0.72) | 0.42 (-0.08 to 0.92) |
| Current job training or other studies | 0.09 (-0.35 to 0.52) | -0.03 (-0.62 to 0.57) | 0.07 (-0.57 to 0.71) |

Abbreviations: β, regression coefficient; 95% CI, 95% Confidence Interval.

^a^ Adjusted models were controlled for age, country of origin, marital status, educational level, and weekly income.

| **eTable 10. Time-varying Cox Regression Models for Weighted Associations of Different Types of Social Participation with Psychological Distress, Stratified by Gender ^a^** | | | |
| --- | --- | --- | --- |
|  | **Hazard ratios (95% CI) ^b^** | | |
|  | **Total** | **Male** | **Female** |
| **Social participation types** |  |  |  |
| Sporting activities (ref=no) | 0.72 (0.56 to 0.93) | 0.65 (0.45 to 0.92) | 0.90 (0.65 to 1.26) |
| Leisure activities (ref=no) | 0.75 (0.57 to 0.98) | 0.70 (0.48 to 1.02) | 0.86 (0.59 to 1.24) |
| School activities (ref=no) | 1.01 (0.79 to 1.29) | 1.17 (0.83 to 1.67) | 0.83 (0.60 to 1.16) |
| Parent support groups (ref=no) | 1.21 (0.86 to 1.70) | 1.06 (0.63 to 1.78) | 1.32 (0.85 to 2.04) |
| Volunteering activities (ref=no) | 1.07 (0.80 to 1.43) | 1.09 (0.72 to 1.65) | 1.08 (0.72 to 1.62) |
| Cultural activities (ref=no) | 1.02 (0.78 to 1.34) | 0.92 (0.60 to 1.39) | 1.14 (0.81 to 1.60) |
| Self-improvement activities (ref=no) | 1.10 (0.81 to 1.49) | 1.25 (0.83 to 1.86) | 0.99 (0.62 to 1.57) |
| Current paid employment status (ref=no) | 0.63 (0.48 to 0.83) | 0.66 (0.46 to 0.94) | 0.68 (0.39 to 1.18) |
| Current English study | 0.98 (0.82 to 1.16) | 0.94 (-0.72 to 1.23) | 0.99 (0.79 to 1.24) |
| Current job training or other studies | 0.90 (-0.70 to 1.16) | 0.98 (0.67 to 1.42) | 0.81 (0.57 to 1.13) |

Abbreviations: 95% CI, 95% confidence Interval.

^a^ Time-varying Cox regression models were weighted using longitudinal weights.

^b^ Adjusted models were controlled for age, country of origin, marital status, educational level, and weekly income.

| **eTable 11. Longitudinal Mixed Linear Models Assessing the Association of Social Participation Diversity with Psychological Distress Scores, Stratified by Gender, Using Imputed Datasets** | | | | | | |
| --- | --- | --- | --- | --- | --- | --- |
| **Diversity of social participation** | **β (95% CI)** | | | | | |
|  | **Total** | | **Male** | | **Female** | |
|  | **Crude model** | **Adjusted model** | **Crude model** | **Adjusted model** | **Crude model** | **Adjusted model** |
| 0 | Reference | Reference | Reference | Reference | Reference | Reference |
| 1 | -1.00 (-1.39 to -0.62) | -0.50 (-0.88 to -0.11) | -1.24 (-1.78 to -0.69) | -0.63 (-1.18 to -0.09) | -0.80 (-1.38 to -0.22) | -0.40 (-1.04 to 0.25) |
| ≥2 | -1.35 (-1.75 to -0.94) | -0.65 (-1.07 to -0.23) | -1.62 (-2.19 to -1.05) | -0.83 (-1.41 to -0.24) | -1.35 (-1.99 to -0.70) | -0.35 (-1.07 to 0.37) |

Abbreviations: β, regression coefficient; 95% CI, 95% Confidence Interval.

^a^ Adjusted models were controlled for age, country of origin, marital status, educational level, and weekly income.

| **eTable 12. Time-varying Cox Regression Models for Unweighted Associations of Social Participation Diversity with Psychological Distress, Stratified by Gender ^a^** | | | | | | | |
| --- | --- | --- | --- | --- | --- | --- | --- |
| **Diversity of social participation** | **Hazard ratios (95% CI)** | | | | | | |
|  | **Total** | | **Male** | | | **Female** | |
|  | **Crude model** | **Adjusted model** | **Crude model** | **Adjusted model** | **Crude model** | | **Adjusted model** |
| 0 | 1.00 (reference) | 1.00 (reference) | 1.00 (reference) | 1.00 (reference) | 1.00 (reference) | | 1.00 (reference) |
| 1 | 0.71 (0.60 to 0.85) | 0.81 (0.67 to 0.99) | 0.56 (0.42 to 0.75) | 0.66 (0.48 to 0.91) | 0.89 (0.71 to 1.12) | | 0.98 (0.76 to 1.26) |
| ≥2 | 0.59 (0.49 to 0.71) | 0.76 (0.61 to 0.95) | 0.53 (0.40 to 0.71) | 0.69 (0.50 to 0.97) | 0.72 (0.56 to 0.93) | | 0.89 (0.66 to 1.20) |

Abbreviations: 95% CI, 95% Confidence Interval.

^a^ Adjusted models were controlled for age, country of origin, marital status, educational level, and weekly income.

**References**

**Department of Social Services & Australian Institute of Family Studies** (2022). Building a New Life in Australia: The Longitudinal Study of Humanitarian Migrants, Release 5 (Waves 1-5): ADA Dataverse.

**Edwards B, Smart D, De Maio J, Silbert M and Jenkinson R** (2018) Cohort Profile: Building a New Life in Australia (BNLA): the longitudinal study of humanitarian migrants. *International Journal of Epidemiology* **47**(1), 20.

**Rioseco P, De Maio J and Hoang C** (2017) The Building a New Life in Australia (BNLA) Dataset: A Longitudinal Study of Humanitarian Migrants in Australia. *The Australian Economic Review* **50**(3), 356-362.

**Wu S, Renzaho A, Hall BJ, Shi L, Ling L and Chen W** (2021) Time-varying associations of pre-migration and post-migration stressors in refugee’ mental health during resettlement: a longitudinal study in Australia. *Lancet Psychiatry* **8**(1), 36-47.
